# Outcome of diaphyseal forearm fracture-nonunions treated by autologous bone grafting and compression plating

**DOI:** 10.1186/1750-1164-3-5

**Published:** 2009-05-18

**Authors:** Fernando Baldy dos Reis, Flávio Faloppa, Hélio J Alvachian Fernandes, Walter Manna Albertoni, Philip F Stahel

**Affiliations:** 1 Department of Orthopaedics and Traumatology, Federal University of Sao Paulo (UNIFESP), Sao Paulo, Brazil; 2 Department of Orthopaedic Surgery, Denver Health Medical Center, University of Colorado School of Medicine, Denver, Colorado, USA

## Abstract

**Background:**

The treatment of forearm fracture-nonunions continues to represent a therapeutic challenge, and reported outcomes are moderate at best. Limiting aspects of this particular anatomic location include the relation between restoration of shaft length with the anatomy and long-term functional outcome of adjacent joints, as well as the risk of elbow and wrist stiffness related to prolonged immobilization. The present study was designed to assess the outcome of autologous bone grafting with compression plating and early functional rehabilitation in patients with forearm fracture non-unions.

**Methods:**

Prospective follow-up study in 31 consecutive patients presenting with non-unions of the forearm diaphysis (radius, *n *= 11; ulna, *n *= 9; both bones, *n *= 11). Surgical revision was performed by restoring anatomic forearm length by autologous bone grafting of the resected non-union from the iliac crest and compression plating using a 3.5 mm dynamic compression plate (DCP) or limited-contact DCP (LC-DCP). The main outcome parameters consisted of radiographic bony union and functional outcome, as determined by the criteria defined by Harald Tscherne in 1978. Patients were routinely followed on a short term between 6 weeks to 6 months, with an average long-term follow-up of 3.6 years (range 2 to 6 years).

**Results:**

Radiographically, a bony union was achieved in 30/31 patients within a mean time of 3.5 months of revision surgery (range 2 to 5 months). Clinically, 29/31 patients showed a good functional outcome, according to the Tscherne criteria, and 26/31 patients were able to resume their previous work. Two postoperative infections occurred, and one patient developed a persistent infected nonunion. No case of postoperative failure of fixation was seen in the entire cohort.

**Conclusion:**

Revision osteosynthesis of forearm nonunions by autologous iliac crest bone grafting and compression plating represents a safe and efficacious modality for the treatment of these challenging conditions.

## Background

The surgical treatment of diaphyseal forearm fracture-nonunions remains a therapeutic challenge for orthopaedic trauma surgeons. Key to success in the management of these demanding conditions is to develop a comprehensive treatment concept which considers the forearm and its adjacent joints, the elbow and wrist, as a complex functional unit [1,2]. Nonunions of the radius and ulna shaft cause a severe anatomic and functional impairment, related to disturbance of the interosseous membrane and dysfunction of the adjacent joints, elbow and wrist [3-6]. These demanding nonunions require the surgical correction to restore the anatomy of the forearm and to improve function [1,7]. New techniques have been recently postulated for the treatment of forearm nonunions, including distraction-compression osteogenesis, locked plating, and locked intramedullary nailing [8-10]. In addition, free fibula transfer flaps have been advocated as a means to restore anatomic length and ensure bony union [11,12].

In the present study, we evaluated the long-term radiological and clinical outcome of 31 consecutive patients treated by autologous bone grafting and compression plating for fracture-nonunions of the forearm. We hypothesized that this "classic" treatment concept would result in excellent clinical outcome and a low incidence of long-term functional impairment.

## Methods

**Figure 1:**
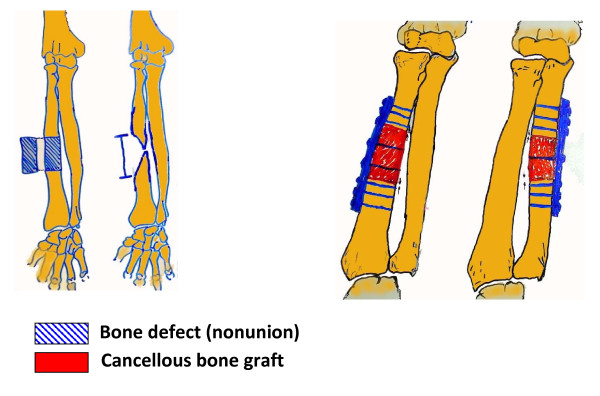
**Preoperative planning scheme for nonunion resection, placement of corticocancellous iliac crest bone graft, and compression plating**.

A retrospective analysis of a prospective database of all consecutive patients treated for fracture-nonunions of the forearm was performed at a single academic center (Dept. of Orthopaedics and Traumatology, Federal University of Sao Paulo, Brazil). The inclusion criteria consisted of all adult patients (> 18 years) of either gender with posttraumatic nonunions of the radius and/or ulna shaft, in absence of an active infection. Patients with nontraumatic forearm pseudarthrosis, infected nonunions, or with an associated neurological impairment of the ipspilateral upper extremity, which may preclude from an adequate functional assessment, were excluded from analysis. All surgical procedures were performed by a single surgeon (F.B.R.). Nonunions were classified according to the standard classification by Weber and Cech [13]. The pre-operative plan included plain X-rays of the forearm, wrist and elbow, in antero-posterior (a.p.) and lateral views. The presence of infection was excluded by preoperative analysis of systemic infection parameters (WBC, sedimentation rate, CRP) and by intraoperative tissue samples of the resected nonunions which were sent for microbiology cultures and histopathological workup. The standardized treatment concept (Figure 1) consisted of resection of the forearm nonunion, autologous bone grafting with a tricortical graft from the iliac crest with anatomic restoration of the foreram length, as determined by intraoperative fluoroscopy assessment of the adjacent joints, and compression plating using a stainless steel small fragment (3.5 mm) dynamic compression plate (DCP) or limited-contact DCP (LC-DCP). Radiological and functional outcome was determined at a minimum of 2 years follow-up, with standardized intervals at 3, 6, 12, and 24 months after revision surgery. The functional outcome was evaluated according to the criteria defined by Tscherne *et al*. [14]. These criteria include the range of motion of forearm pro-/supination, wrist and elbow flexion and extension, ulnar shift/deviation, and positive/negative ulnar variant on wrist X-rays. Bony union was defined in the presence of a minimum of three bridging cortices in plain X-rays of the forearm in a.p. and lateral views, in absence of pain at the nonunion site.

## Results and discussion

**Figure 2:**
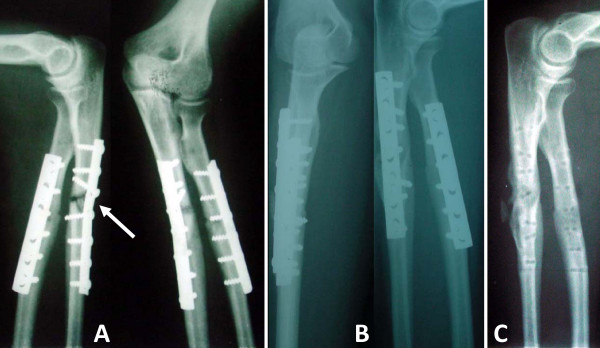
**Clinical case example of a patient with a fracture-nonunion of the ulna shaft secondary to an unstable treatment modality using a small fragment tubular plate, lacking adequate stability with interfragmentary compression (A)**. Revision surgery was performed by corticocancellous bone grafting and compression plating using a more rigid construct with a dynamic compression plate (**B**). Panel **C **shows the fully healed radius fracture and ulnar fracture-nonunion after hardware removal.

A total of 32 consecutive patients were available for prospective enrollment. One patient died from a cardiovascular condition (myocardial infraction) in the postoperative course and was therefore lost to follow-up. The remaining 31 patients (27 males and 4 females; median age 30 years) presenting with forearm fracture-nonunions were prospectively enrolled into this study. Of these, 26 patients had their dominant arm affected, and 8 patients had a previous history of infection. Eleven patients presented with a both bone fracture-nonunion, and 20 patients had a single bone affected (11 radius, 9 ulna shaft). Eight patients had a history of a previous local infection, which however was completely healed at the time of revisions surgery, as determined by negative intraoperative bone biopsies and tissue cultures. The number of previous surgeries on the affected forearm varied from 1 to 5 (median 1.5). Patients presented with a fracture-nonunion within 5 to 24 months after the initial surgery (median of 7.5 months). According to the Weber and Cech classification, 28 cases (90%) were defined as atrophic, nonviable nonunions. Fourteen of these 28 patients had a segmental bone defect ranging from 1 to 5 cm (median 2.3 cm). Radiographically, a bony union was achieved in 30/31 patients within a mean time of 3.5 months of revision surgery (range 2 to 5 months). Clinically, 29/31 patients showed a good functional outcome, according to the Tscherne criteria, and 26/31 patients were able to resume their previous work. An illustrative case example of uneventful nonunion healing is shown in figure 2.

Two patients developed a posteroperative infection, of which one case was successfully managed by surgical debridement and antibiotics for two weeks. The other patient developed a persistent infected nonunion requiring further revision surgery. This was the only case of the entire cohort of 31 patients which failed to achieve bony union. No postoperative failure of fixation was seen in any patient. One patient was lost to long-term follow-up secondary to death related to a cardiovascular event at 5 months, when the nonunion was fully healed. No association was shown between the time of bony union and the time elapsed between the trauma and the last surgery prior to presentation with a nonunion (data not shown). Residual radiological or clinical deformities were observed in 13 patients (table 1). These radiological findings did not appear to significantly influence the functional outcome, based on the observed clinical outcome in 29/31 patients, as defined by the Tscherne criteria, with less than 10% of restricted range of motion at the wrist and elbow (table 2). Regarding forearm rotation (pro-/supination), 17/31 patients presented losses lower than 20 degrees, and 9/31 presented moderate results with pro/-supination loss between 20–40 degrees. A total of 26/31 patients were able to resume their previous work.

**Table 1:** Clinical and radiological residual deformities in 13 patients following revision surgery for forearm fracture-nonunions

Residual deformity	Number of patients (total n = 13)
Positive ulnar variant: 1 mm	4
Positive ulnar variant: 2 mm	1
Negative ulnar variant: 1 mm	2
Ulnar head prominence	2
Ulnar head absence	1
Loss of radial bow	3

**Table 2:** Functional outcome in 30 patients with radiologically healed forearm fracture nonunions, assessed 6 months after revision surgery

ROM* wrist	Number of patients
Normal (identical to contralateral side)	26
Flexion/extension 90°/0°/60°	3
Limited ulnar shift	1
	
ROM* elbow	Number of patients

Normal (identical to contralateral side)	27
Extension/flexion 0°/20°/120°	1
Extension/flexion 0°/30°/120°	2
	
Forearm rotation	Number of patients

Normal (identical to contralateral side)	17
Pro-/supination 90°/0°/60°	5
Pro-/supination 90°/0°/45°	3
Pro-/supination 80°/0°/70°	1
Pro-/supination 60°/0°/45°	1
Pro-/supination 45°/0°/30°	3

*ROM, range of motion.

This study demonstrates the efficacy and safety for the treatment concept of autologous bone grafting and compression plating for forearm fracture-nonunions, leading to excellent radiological and functional long-term outcome. Reconstruction of the anatomy of both forearm bones is of crucial importance in the management of the diaphyseal forearm nonunions [1-6]. The concept of corticocancellous iliac crest bone grafting and compression plating for, was previously postulated as an early treatment strategy for traumatic segmental defects of the upper extremity, including forearm fractures [15]. Despite open wounds in some patients which healed by secondary intention, the exposed cortical bone graft was not shown to be prone to infection [15]. More recently, a retrospective analysis of 41 patients with comminuted both bone forearm fractures treated by compression plating with or without primary bone grafting determine a nonunion rate of 12%, and no benefit was revealed for early bone grafting with regard to the rate of union [16]. Barbieri and colleagues reported their experience in a case series of 12 patients treated by iliac crest bone block grafting and compression plating for diaphyseal defects of the forearm, secondary to infection and bone loss [17]. The authors demonstrated a successful union in 10/12 patients, within a mean time period of 17 weeks after the surgical revision [17]. However, a high rate of 30% recurrent infections of was reported in this cohort, which questions the safety of autologous bone grafting in the setting of posttraumatic infection and chronic osteomyelitis. Similarly, Moroni and colleagues reported a high incidence of infection of 12.5% after intercalary bone graft fixation in patients with isolated forearm nonunions [18]. In the present study, the incidence of postoperative infection was much lower (2/31 patients). One case resulted in failure by developing a chronic infected nonunion, while the other case was successfully managed by surgical debridement and antibiotic therapy, resulting in a healed union and a good functional long-term outcome. Bony union were achieved in 96.7% of all cases (30/31) on average time of 3.5 months. The functional outcome measured by the Tscherne's criteria showed good results in 26/31 patients. Based on these findings, our data confirm the safety and efficacy of autologous bone grafting and compression plate fixation of fracture nonunions of the forearm [19].

## Conclusion

Autogenous cortical bone grafts were historically described as a successful modality for the reconstruction of traumatic segmental skeletal defects [20-23]. While the plate fixation of forearm fractures remains the gold standard, complications have been shown to occur in up to 28% of all patients [24]. One of the major challenges of long-term complications are forearm nonunions with bone loss and segmental defects. In the present study, we demonstrate the safety and efficacy of corticocancellous iliac crest bone grafting and compression plating for revision fixation of forearm fracture-nonunions, leading to excellent radiological and functional long-term outcomes.

## Competing interests

The authors declare that they have no competing interests.

## Authors' contributions

FBR, FF, HJAF, and WMA designed the study and were responsible for the clinical care of the patients. FBR and PFS wrote and edited the manuscript. All authors read and approved the final version of this paper.

## References

[B1] Rochard MJ, Ruch DS, Aldridge JM (2007). Malunions and nonunions of the forearm. Hand Clin.

[B2] Richards RR (1996). Chronic disorders of the forearm. J Bone Joint Surg (Am).

[B3] Schemitsch EH, Richards RR (1992). The effects of malunion on functional outcome after plate fixation of fractures of both bones of the forearm in adults. J Bone Joint Surg (Am).

[B4] Hollister AM, Gellman H, Waters RL (1994). The relationship of the interosseous membrane to the axis of rotation of the forearm. Clin Orthop Relat Res.

[B5] Skahen JR, Palmer AK, Werner FW, Fortino MD (1997). The interosseous membrane of the forearm: anatomy and function. J Hand Surg (Am).

[B6] Tarr RR, Garfinkel AI, Sarmiento A (1984). The effects of angular and rotational deformities of both bones of the forearm. J Bone Joint Surg (Am).

[B7] Jupiter JB, Rüedi T (1992). Intraoperative distraction in the treatment of complex nonunions of the radius. J Hand Surg (Am).

[B8] Orzechowski W, Morasiewicz L, Dragan S, Krawczyk A, Kulej M, Mazur T (2007). Treatment of non-union of the forearm using distraction-compression osteogenesis. Ortop Traumatol Rehabil.

[B9] Ling HT, Kwan MK, Chua YP, Deepak AS, Ahmad TS (2006). Locking compression plate: a treatment option for diaphyseal nonunion of radius or ulna. Med J Malaysia.

[B10] Krzykawski R, Król R, Kamiński A (2008). The results of locked intramedullary nailing for non-union of forearm bones. Ortop Traumatol Rehabil.

[B11] Safoury Y (2005). Free vascularized fibula for the treatment of traumatic bone defects and nonunion of the forearm bones. J Hand Surg [Br].

[B12] Saint-Cyr M, Farkas J, Gupta A (2008). Double-barrel free fibula flap for treatment of infected nonunion of both forearm bones. J Reconstr Microsurg.

[B13] Weber BG, Cech O (1975). Pseudarthrosis of the forearm. Pseudarthrosis.

[B14] Tscherne H, Oestern HJ, Sander U (1978). Technique and results of rigid-plate fixation in forearm fractures. Unfallheilkunde.

[B15] Calkins MS, Burkhalter W, Reyes F (1987). Traumatic segmental bone defects in the upper extremity. Treatment with exposed grafts of corticocancellous bone. J Bone Joint Surg (Am).

[B16] Ring D, Rhim R, Carpenter C, Jupiter JB (2005). Comminuted diaphyseal fractures of the radius and ulna: does bone grafting affect nonunion rate?. J Trauma.

[B17] Barbieri CH, Mazzer N, Aranda CA, Pinto MM (1997). Use of a bone block graft from the iliac crest with rigid fixation to correct diaphyseal defects of the radius and ulna. J Hand Surg [Br].

[B18] Moroni A, Rollo G, Guzzardella M, Zinghi G (1997). Surgical treatment of isolated forearm nonunion with segmental bone loss. Injury.

[B19] Ring D, Allende C, Jafarnia K, Allende BT, Jupiter JB (2004). Ununited diaphyseal forearm fractures with segmental defects: plate fixation and autogenous cancellous bone grafting. J Bone Joint Surg [Am].

[B20] Miller RC, Phalen GS (1947). The repair of defects of the radius with fibular bone grafts. J Bone Joint Surg [Am].

[B21] Dabezies EJ, Stewart WE, Goodman FG, Deffer PA (1971). Management of segmental defects of the radius and ulna. J Trauma.

[B22] Enneking WF, Eady JL, Burchardt H (1980). Autogenous cortical bone grafts in the reconstruction of segmental skeletal defects. J Bone Joint Surg [Am].

[B23] Grace TG, Eversmann WW (1980). The management of segmental bone loss associated with forearm fractures. J Bone Joint Surg [Am].

[B24] Stern PJ, Drury WJ (1983). Complications of plate fixation of forearm fractures. Clin Orthop Relat Res.

